# The study on analysis of risk factors for severity of white matter lesions and its correlation with cerebral microbleeds in the elderly with lacunar infarction

**DOI:** 10.1097/MD.0000000000018865

**Published:** 2020-01-24

**Authors:** Yu-Ni Zhou, Hao-Yuan Gao, Fang-Fang Zhao, Ying-Chun Liang, Yuan Gao, Xin-Hong Liu, Tao Wang, Zhi-Gao Wang, Qing-Jian Wu

**Affiliations:** a Department of Neurology, Jining Psychiatric Hospital, Jining; b Department of Cardiology, The Second Affiliated Hospital of Shandong First Medical University; c Department of Neurology, Tai’an City Central Hospital, Tai’an; d Department of Psychiatry, Jining Psychiatric Hospital, Jining, Shandong; e Department of Statistics, East China Normal University, Shanghai; f Department of Emergency, Jining NO.1 People's Hospital, Jining, Shandong, China.

**Keywords:** cerebral microbleeds, deep white matter lesions, Fazekas score, lacunar infarction, ventricular paraventricular white matter lesions, white matter lesions

## Abstract

This study aimed to explore the risk factors for severity of white matter lesions and its correlation with in the elderly with lacunar infarction.

Patients (range, 70–85 years) with lacunar infarction treated in a hospital in China from 2016 to 2017were enrolled. Fazekas rating scale (0–6 points) was used to assess severity of white matter lesions. Risk factors for the severity of white matter lesions and correlation between cerebral microbleeds and white matter lesions in the elderly with lacunar infarction were studied.

The elderly (81–85 years’ old, odds ratio [OR]: 2.423, 95% confidence interval [CI]: 1.795–3.271, *P* = .018; 76∼80 years’ old, OR: 3.113, 95% CI: 1.723–5.625, *P* = .043), carotid atherosclerosis (OR: 3.062, 95% CI:1.715–5.468, *P* < .001), history of hypertension (OR: 3.694, 95% CI: 2.031–6.717, *P* < .001) were risk factors for the severity of white matter lesions. The white matter lesions score increased corresponding to increase in the cerebral microbleeds grade (*P* < .001). The white matter lesions score was higher in the cerebral microbleeds combined with the white matter lesions group than in the white matter lesions group (*P* < .01). After correcting the effects of age, there was a correlation between white matter lesions and cerebral microbleeds (*P* < .001). Logistic analysis revealed that the patients’ age (81–85 years’ old, OR: 2.722, 95% CI: 1.985–3.734, *P* = .019; 76∼80 years’ old, OR: 1.857, 95% CI: 1.075–3.207, *P* = .031), history of hypertension (OR: 2.931, 95% CI: 1.136–7.567, *P* = 0.0.036), systolic blood pressure (OR: 1.049, 95% CI: 1.015–1.084, *P* = .007), high-sensitivity C-reactive protein (OR: 1.504, 95% CI: 1.254–1.803, *P* < .001), homocysteine (OR: 1.076, 95% CI: 1.020–1.136, *P* = .009), and carotid atherosclerosis (OR: 1.389, 95% CI: 1.103–1.748, *P* = .010) were significant risk factors for combined cerebral microbleeds with white matter lesions in patients with lacunar infarction.

The elderly, carotid atherosclerosis, history of hypertension were risk factors for the severity of white matter lesions. Cerebral microbleeds were positively correlated with the severity of white matter lesions.

## 1 Introduction

Currently, ischemic stroke is a severe global problem with high recurrence rate that imposes severe human-health burden.^[1]^ In China, lacunar infarction accounts for about 25% of ischemic stroke. Cerebral microbleeds (CMBs) are a type of cerebral small vessel disease. It refers to the deposition of hemosiderin and ferritin around the tiny blood vessels where leakage or rupture occurs, which in turn impairs the brain parenchyma. It is more common in the cortical–cortical and deep brain tissues. CMBs in the susceptibility weighted imaging (SWI) showed a diameter of <10 mm, and there is no edema around.^[2]^ The incidence of CMBs in lacunar infarction was 27%.^[3]^ White matter lesions (WML) are also a category of cerebral small vessel disease. WML occurs mostly in the elderly population, and Inzitari et al^[4]^ showed that the incidence of WML was 24% in stroke patients aged 60 to 85 years. The pathogenesis of CMBs and WML is related to destruction of the blood–brain barrier,^[5,6]^ with many risk factors for disease onset. At present, there are still relatively few reports on risk factors affecting the severity of WML and the relationship between CMBs and WML is unclear. This study explored risk factors for severity of WML and its correlation with CMBs in the elderly with lacunar infarction.

## 2 Materials and methods

### 2.1 Study subjects

Patients with lacunar infarction who were admitted to the Neurology Department of the Tai’an Central Hospital and hospitalized from September 2016 to November 2017 were included. T1-weighted imaging (T1WI), T2-weighted imaging (T2WI), fluid-attenuated inversion recovery (FLAIR), and susceptibility-weighted imaging (SWI) were performed. According to the results of previous clinical studies, at the level of α = 0.05, to achieve 90% of the test efficacy, a total of 175 patients were enrolled. According to the results of brain magnetic resonance imaging (MRI), the patients were divided into the WML group and non-WML group. Fazekas et al's^[7]^ rating scale (0–6 points) was used to assess severity of WML. The periventricular hyperintensities (PVH) and the deep white matter hyperintensities (DWMH) were scored separately and subsequently added as the total score. Overall, the grading was as follows. Cerebral ventricular high-signal score: 0 points, no high-signal lesions; 1 point, cap-like or pencil-thin-like lesions; 2 points, lesions showing smooth halos; and 3 points, high-signal irregularities around the ventricles. DWMH: 0 points, no high-signal lesions; 1 point, spotted lesions; 2 points, lesions beginning to fuse; and 3 points, lesions showing large-area fusion. With regard to the total score, the score was divided into 1 (1), 2 (2–3), and 3 (4–6); and per severity of CMBs, it was divided into 0 to 3 grades: Grade 0, absence of CMBs lesion; grade 1, 1 to 5 lesions; grade 2, 6 to 10 lesions; and grade 3, >10 lesions. Inclusion criteria were as follows: lacunar infarction patients who met the diagnostic criteria per 2010 Guidelines for the Prevention and Treatment of Acute Ischemic Stroke in China^[8]^ and were confirmed through brain MRI and carotid ultrasound examination; aged 70 to 85 years. All patients or their family members signed the informed consent form. The study was approved by the hospital's Ethics Committee. Exclusion criteria were as follows: acute large area cerebral infarction, acute cerebral hemorrhage or previous cerebral hemorrhage history; serious heart, liver, and kidney organ dysfunction; pregnant women; traumatic brain injury.

### 2.2 Assessment of carotid AS

We assessed carotid Atherosclerosis by a Color Doppler ultrasound system (Aplio XG SSA-790A; Toshiba Medical Systems Corporation, Tokyo, Japan), including carotid intima-media thickness (CIMT) and plaque Crouse score. The judgment criteria are that in longitudinal sections, CIMT value: ≤1.0 mm was considered to be healthy, >1.0 mm indicates the formation of carotid atherosclerosis. We used Crouse scoring to assess plaques. The judgment criterion of Crouse scoring is the maximum thickness (millimeter) of each plaque. The sum of the bilateral maximum CIMT values was calculated to determine Crouse score. If the CIMT value ≤1.2 mm, the Crouse score was 0. Physicians who were unaware of the clinical data recorded Carotid ultrasonography data.

### 2.3 Head MRI examination

In this study, MRI was conducted using Siemens Magnetom Skyra 3.0 T superconducting MR (Siemens, Erlangen, Germany), and head coil was used. All patients underwent spin-echo sequence T1WI, T2WI, FLAIR, and SWI with the following scan parameters. T1WI: TR 2000 ms, TE 32 ms, FOV 240 mm, and layer thickness 5 mm; T2WI: TR 6000 ms , TE 125 ms; FOV 240 mm; and layer thickness, 5 mm. FLAIR: TR, 8500 ms; TE, 102 ms; FOV 240 mm; and layer thickness 5 mm. SWI: TR 27 ms; TE 20 ms; FOV 220 mm; and layer thickness 1.5 mm. In all patients, the acquired images were read by 2 experienced physicians in the imaging department, and related image data were recorded.

### 2.4 Statistical analysis

Statistical Package for the Social Sciences (SPSS) version 22.0 software (SPSS Inc., Chicago, IL) was used. In data conforming to normal distribution application 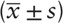, *t* test was used for comparison between the 2 groups, and variance analysis was used for that between multiple groups. A rank comparison test was used for comparison between groups; data were expressed as frequency (or percentage), and *χ*^2^ test was applied for comparison between groups. A logistic regression model was used to analyze the related factors of severity of WML and combined CMBs with WML using a forward stepwise selection strategy. *P* value of <.05 was considered as significant difference.

## 3 Results

### 3.1 Comparison of baseline clinical data according to different degrees of WML in patients with lacunar infarction

In this study, there were 130 cases of WML and 45 cases of no WML in patients with lacunar infarction. Patients were divided into 4 groups according to the degree of WML. The patients’ age, history of hypertension, and indicators of carotid atherosclerosis (Carotid intima-media thickness [CIMT], Crouse score) were significantly different in patients with different degrees of WML (*P* < .05), as shown in Table 1.

**Table 1:**
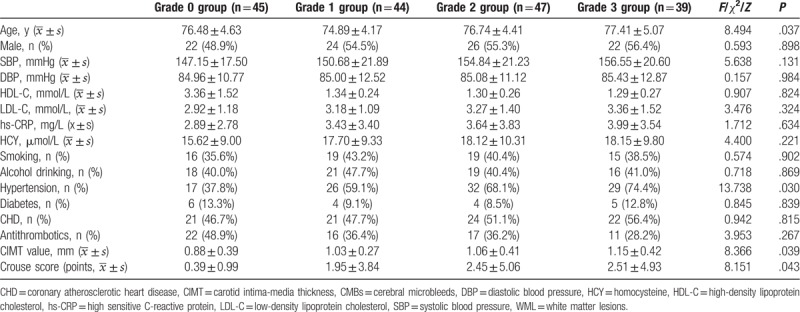
Comparison of baseline data among the different groups of WML.

### 3.2 Logistic regression analysis of ordered multiclassification of severity of WML

The severity of WML was the dependent variable (Grade 0 = 1, grade 1 = 2, grade 2 = 3, grade 3 = 4). Significant variables in univariate analysis, such as age, history of hypertension, arteriosclerosis, and according to previous studies, variables, which may be statistically significant such as sex, homocysteine (HCY), and baseline systolic blood pressure, were independent variables. The age of the patient is divided into 3 age groups, 70 to 75 years’ old, 76 to 80 years’ old, 81 to 85 years’ old. The results showed that the elderly (81–85 years’ old, odds ratio [OR]: 2.423, 95% confidence interval [CI]: 1.795–3.271, *P* = 0.018; 76∼80 years’ old, OR: 3.113, 95% CI: 1.723–5.625, *P* = 0.043), carotid atherosclerosis (OR: 3.062, 95% CI:1.715–5.468, *P* < .001), history of hypertension (OR: 3.694, 95% CI: 2.031–6.717, *P* < .001) were risk factors for the severity of WML (Table 2).

**Table 2:**
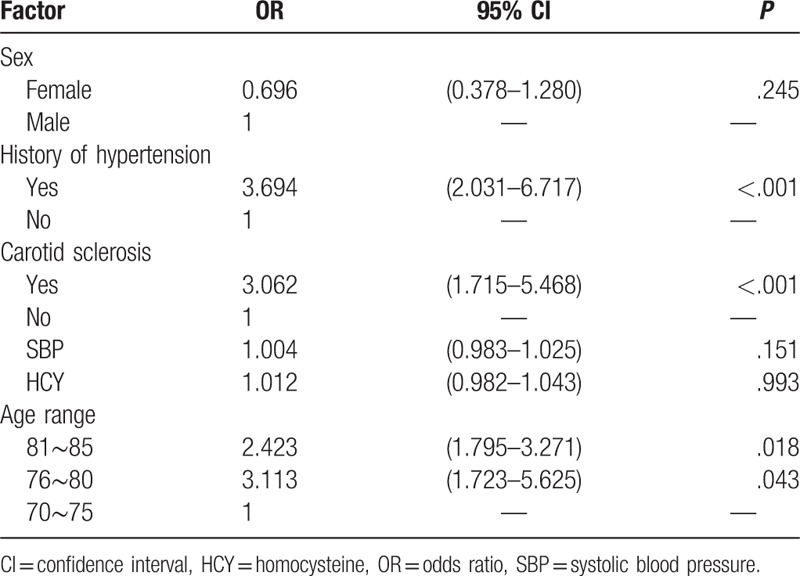
Logistic regression analysis of ordered multi-classification of severity of white matter lesions.

### 3.3 Relationship between the CMBs’ grade and WMLs’ score

**Figure 1:**
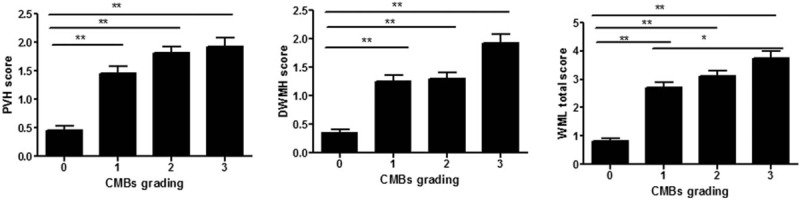
Comparison of PVH score, DWMH score, and WML total score in different grades of CMBs. (A) The PVH score increased with the severity of CMBs. The comparison between the 2 groups showed that the difference between CMBs0 and CMBs1, CMBs0 and CMBs2, CMBs0 and CMBs3 was statistically significant. (B) The DWMH score increased with the severity of CMBs. The comparison between the 2 groups showed that the difference between CMBs0 and CMBs1, CMBs0 and CMBs2, CMBs0 and CMBs3 was statistically significant. (C) The WML total score increased with the severity of CMBs. The comparison between the 2 groups showed that the difference between CMBs0 and CMBs1, CMBs0 and CMBs2, CMBs0 and CMBs3, CMBs1 and CMBs3 was statistically significant. ^∗^*P* < .05,^∗∗^*P* < .01. Comparison of PVH score, DWMH score, and WML total score in different grades of CMBs. CMBs = cerebral microbleeds, DWMH = deep white matter hyperintensities, PVH = periventricular hyperintensities, WML = white matterlesions.

In patients with lacunar infarction, according to the severity of CMBs, there were 4 groups of 0 to 3. And the PVH score, DWMH score, and WML total score of patients in different degrees of CMBs were compared. The PVH score increased with the severity of CMBs. There was a statistically significant difference in PVH scores of patients in different grades of CMBs (*P* < .001). The comparison between the two groups showed that the difference between CMBs0 and CMBs1, CMBs0 and CMBs2, CMBs0 and CMBs3 was statistically significant (*P* < .01). The DWMH score increased with the severity of CMBs. There was a statistically significant difference in DWMH scores of patients in different grades of CMBs (*P* < .001). The comparison between the 2 groups showed that the difference between CMBs0 and CMBs1, CMBs0 and CMBs2, CMBs0 and CMBs3 was statistically significant (*P* < .05). WML total score increased with the severity of CMBs. There was a statistically significant difference in WML total scores of patients in different grades of CMBs (*P* < .001). The comparison between the 2 groups showed that the difference between CMBs0 and CMBs1, CMBs0 and CMBs2, CMBs0 and CMBs3, CMBs1 and CMBs3 was statistically significant (*P* < .01) (Fig. 1).

### 3.4 Comparison of PVH score, DWMH score, and WML total score in patients with WML group and CMBs combined with WML group

**Figure 2:**
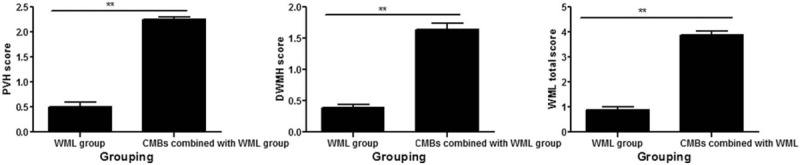
Comparison of PVH score, DWMH score and WML total score in patients with WML group and CMBs combined with WML group. The A, B, and C graphs show that the PVH score, DWMH score, and WML total score are higher in the CMBs combined with the WML group than in the WML group.^∗∗^*P* < 0.01. CMBs = cerebral microbleeds, DWMH = deep white matter hyperintensities, PVH = periventricular hyperintensities, WML = white matterlesions.

In patients with lacunar infarction, we screened WML patients and CMBs combined with WML patients, and the PVH score, DWMH score, and WML total score were compared respectively between the 2 groups. The results of the study showed that the PVH score was higher in the CMBs combined with the WML group than in the WML group, and the difference was statistically significant (*P* < .001). The DWMH score was higher in the CMBs combined with the WML group than in the WML group, and the difference was statistically significant (*P* < .001). The WML total score was higher in the CMBs combined with the WML group than in the WML group, and the difference was statistically significant (*P* < .001) (Fig. 2).

### 3.5 Comparison of the relationship between the WML score and the average number of CMBs

**Figure 3:**
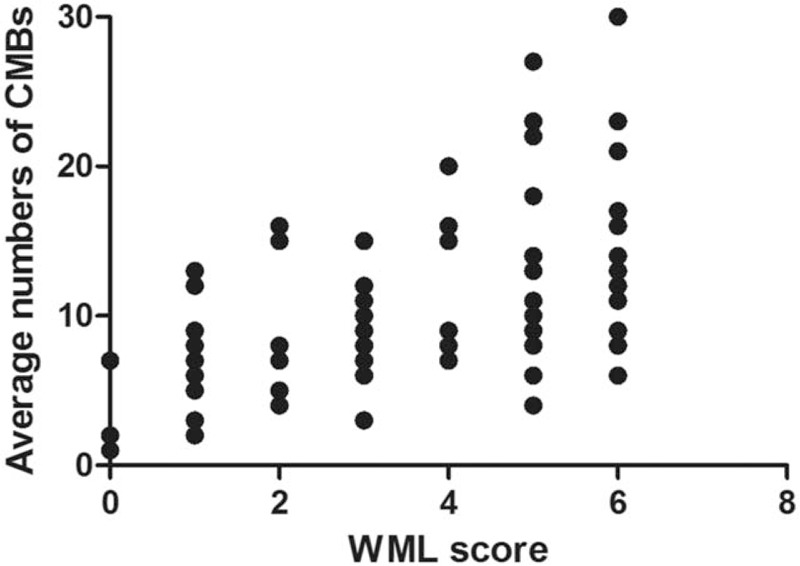
Scatter plot of comparison of the relationship between the WML score and the average number of CMBs. CMBs = cerebral microbleeds, WML = white matterlesions.

In a scatter plot, considering WML score as abscissa and number of CMBs as ordinate (Fig. 3), the average value of the CMBs’ number increased corresponding to an increase in the WML score. And there was a correlation between the two (*r* = 0.537, *P* < .001).

### 3.6 Partial correlation analysis between WML and CMBs

After correcting the effects of age, the results of this study showed that there was a correlation between WML grade and CMBs grade, total WML score and CMBs’ number, total WML score and CMBs grade, WML grade, and CMBs’ number, with r-value of 0.557, 0.569, 0.570, and 0.613, respectively, and each *P* < .001 indicating statistically significant differences, as shown in Table 3.

**Table 3:**
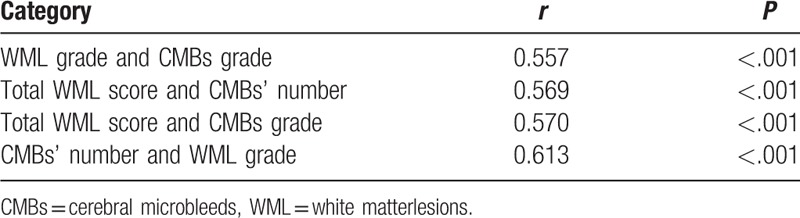
Partial correlation analysis between WML and CMBs.

### 3.7 Analysis of risk factors in patients with combined CMBs with WML

Among patients with lacunar infarction, logistic regression analysis was conducted with or without CMBs combined with the WML as the dependent variable (yes = 1, no = 0). Significant variables in univariate analysis, such as age, history of hypertension, arteriosclerosis, and according to previous studies, variables which may be statistically significant such as sex, coronary heart disease, hyperlipidemia and antithrombotic use, systolic blood pressure, diastolic blood pressure, high-sensitivity C-reactive protein (hs-CRP), HCY were used as independent variables. Logistic analysis revealed that the patients’ age (81–85 years’ old, OR: 2.722, 95% CI: 1.985–3.734, *P* = 0.019; 76∼80 years’ old, OR: 1.857, 95% CI: 1.075–3.207, *P* = 0.031), history of hypertension (OR: 2.931, 95% CI: 1.136–7.567, *P* = 0.0.036), systolic blood pressure (OR: 1.049, 95% CI:1.015–1.084, *P* = .007), hs-CRP (OR: 1.504, 95% CI: 1.254–1.803, *P* < .001), HCY (OR: 1.076, 95% CI: 1.020–1.136, *P* = .009), and carotid atherosclerosis (OR: 1.389, 95% CI: 1.103–1.748, *P* = 0.010) were significant risk factors for combined CMBs with WML in patients with lacunar infarction (Table 4).

**Table 4:**
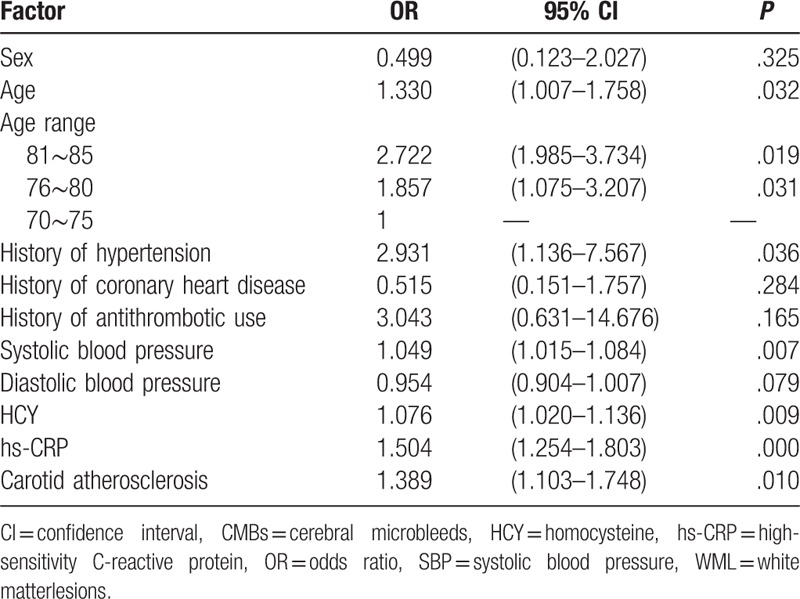
Logistic regression analysis of relevant risk factors for WML and CMBs in patients with lacunar infarction (OR, 95% CI).

## 4 Discussion

With advances in the development of clinical imaging, understanding of cerebral small vessel disease (CSVD) is improving. The International Stroke Conference and the European Stroke Conference of 2008 proposed the concept that small vascular disease causes big trouble.^[9]^ CSVD is a brain lesion caused by cerebral arterioles, venules, and capillary lesions. WML and CMBs belong to the CSVD type. CMBs is hemorrhagic change of CSVD, which is manifested by the deposition of hemosiderin; whereas, WML is ischemic change of CSVD, which shows the loss of brain cells and myelin. Clinically, we found that CMBs are often associated with WML. WML and CMBs are common in the elderly population. The combined occurrence of WML and CMBs is particularly troublesome for patient management involving antithrombotic drugs in clinical first- and second-level prevention of stroke and the factors that influence the development of both are essential for preventing the occurrence of related diseases.

The results from analyses of clinical baseline data at different levels of WML indicated that the patients’ age, history of hypertension, carotid atherosclerosis index CIMT, and Crouse score were significantly different between the groups with different degrees of WML (*P* < .05). Studies have shown that the severity of WML increases with the patients’ age, an increase of the proportion of hypertension history, and aggravation of carotid atherosclerosis the degree. Moreover, logistic regression analysis of ordered multiclassification of severity of WML revealed that the elderly, carotid atherosclerosis history of hypertension were risk factors for the severity of WML. Grueter and Schulz and Simpson et al's^[10,11]^ studies have shown that age and hypertension are important risk factors for WML in patients with ischemic stroke. Also, for every 10 years of age, the incidence of WML increases by a factor of 2 to 3,^[12]^ since myelin necessary protein and lecithin, which constitute the myelin sheath of the white matter, are produced at an early age and show decreasing levels in adults through the aging process. Veerhaaren et al^[13]^ showed that the severity of hypertension was positively correlated with the occurrence of WML. Hypertension aggravated the progression of WML, and high blood pressure, and blood pressure circadian rhythm, and its variability affected the development of WML. This study showed that the increasing severity of carotid atherosclerosis results in an increasing degree of WML; however, the relationship between carotid atherosclerosis and WML is controversial. Patankar et al^[14]^ reported that macrovascular disease has a protective effect on distal small blood vessels, thereby reducing the occurrence of WML. However, some studies said that carotid atherosclerosis was unrelated to the occurrence of WML.^[15]^ This study did not indicate significant differences in sex between the WML group and the control group in patients with lacunar infarction, which is inconsistent with most reports. Most reports outside of China suggested that women were more likely to have WML than men,^[16]^ possibly due to changes in the hormone levels. Estrogen has a protective effect on the brain cells and can reduce their sensitivity to hypoxia. In postmenopausal women, the level of estrogen declines, which may lead to ischemia of the white matter and hypoxia.

In this study, CMBs were classified into 0 to 3 grades according to their number, and WML was classified into 0 to 3 grades according to the WML score too. The results showed that in a scatter plot, the average value of the CMBs’ number increased corresponding to increase in the WML score (*r* = 0.569, *P* < .001). As the severity of CMBs increased, the score of PVH, the score of DWMH, and the total score of WML also increased. The score of PVH, the score of DWMH, and the total score of WML were higher in the CMBs combined with the WML group than in the WML group, and the difference was statistically significant (*P* < .001). Partial correlation analysis between WML and CMBs showed that the severity of CMBs was positively correlated with WML. Yamada et al^[17]^ reported that WML and arteriosclerosis were risk factors for CMBs, and the number of CMBs was correlated with the severity of PVH and the severity of DWMH. Igose et al^[18]^ reported greater severity of PVH in patients with CMBs versus the control group, and positive correlation between the severity of CMBs and the PVH. Thus, the severity of CMBs was positively correlated with the severity of WML. But why is there such a correlation between them? We further explore the pathological manifestations and pathogenesis of the two. With regard to associated pathological changes of CMBs and WML, CMBs is characterized by deposition of hemosiderin around the diseased blood vessels, and WML by the demyelinated nerve fibers caused by gliosis, whereas in case of combined occurrence of both conditions, disruption of the blood-brain barrier can lead to CMBs,^[6,19]^ as well as WML,^[5]^ and hypertension-induced vascular hyalinosis, arteriosclerosis are risk factors for CMBs^[20]^ and WML.^[13]^ Further study is needed to determine whether these are common pathways. In addition, further discussion is also needed, whether there is other pathogenesis.

To demonstrate the risk factors associated with CMBs and WML, 2-class logistic regression analysis was performed. As a result, the patients’ age history of hypertension, systolic blood pressure, hs-CRP, HCY, and carotid atherosclerosis were risk factors for WML combined with CMBs in patients with lacunar infarction. In patients with lacunar infarction, there are many common risk factors for CMBs combined with WML, which suggests that CMBs and WML have different pathological results under the same elements. The combined occurrence of both conditions can increase the risk of ischemic stroke, as well as lead to cerebral hemorrhage. A study has shown that patients with higher CMBs’ number were more likely to have cerebral hemorrhage.^[21]^ Biffi et al^[22]^ reported that in patients with cerebral hemorrhage, the detection rate of WML was increased. Another study^[23]^ reported an increased detection rate of cerebral hemorrhage in patients with WML, suggesting that combined CMBs with WML are associated with risk of cerebral hemorrhage. Therefore, clinicians should pay close attention to the possible occurrence of cerebral hemorrhage in patients with ischemic stroke with combined CMBs with WML undergoing thrombolysis and antithrombotic therapy. Characteristics of combined CMBs with WML showed potential as an early warning sign of cerebral hemorrhage.

In summary, there was a significant correlation between CMBs and WML in the elderly with lacunar infarction, and the severity of the 2 was positively correlated. Risk factors were consistent between CMBs and WML. Except for the patients’ age, which is an uncontrollable factor, several of the identified factors can reverse or delay the development of disease through early detection, diagnosis, and treatment, and consequently prevent brain injury. Improved understanding of the occurrence of vascular complications improves the patients’ quality of life and allows future evidence-based prevention and treatment of patients with CSVD in clinical practice. There are some deficiencies in this study. First, the sample size is small, and second, the selected population is limited in the elderly with lacunar infarction. Therefore, future research needs to be carried out in large sample size and in a wider population.

## Author contributions

**Conceptualization:** Hao-yuan Gao, Fang-fang Zhao, Qing-jian Wu.

**Data curation:** Hao-yuan Gao, Zhi-gao Wang, Qing-jian Wu.

**Formal analysis:** Qing-jian Wu.

**Investigation:** Fang-fang Zhao, Ying-chun Liang, Yuan Gao, Xin-hong Liu, Qing-jian Wu.

**Methodology:** Fang-fang Zhao, Ying-chun Liang, Yuan Gao, Xin-hong Liu, Tao Wang, Qing-jian Wu.

**Project administration:** Fang-fang Zhao, Ying-chun Liang, Tao Wang, Qing-jian Wu.

**Resources:** Qing-jian Wu.

**Supervision:** Xin-hong Liu, Qing-jian Wu.

**Writing – original draft:** Yu-ni Zhou.

**Writing – review & editing:** Yu-ni Zhou, Hao-yuan Gao.
